# Factors predicting self-care behavior of cardiovascular patients during the COVID-19 epidemic

**DOI:** 10.1186/s12872-024-03882-3

**Published:** 2024-05-09

**Authors:** Naser Sharafkhani, Mostafa Yousefi Ghaleh Gazhdomi, Samaneh Norouzi, Mahmoud Ghasemi, Arash Salahshouri

**Affiliations:** 1 Department of Health Education & Health Promotion, Mahabad Faculty of Medical Sciences, Urmia University of Medical Sciences, Urmia, Iran; 2 Department of Oncology and Radiotherapy, Golestan Teaching Hospital of Ahvaz, Ahvaz, Iran; 3 Department of Health Education and Promotion, Faculty of Medical Sciences, Tarbiat Modares University, Tehran, Iran; 4 Institute of Public Health and Clinical Nutrition, Faculty of Health Sciences, The University of Eastern Finland, P.O. Box 1627, Kuopio, 70211 Finland; 5 Social Determinants of Health Research Center, Ahvaz Jundishapur University of Medical Sciences, Ahvaz, Iran; 6 Department of Health Education and Promotion, School of Health, Ahvaz Jundishapur University of Medical Sciences, Ahvaz, Iran

**Keywords:** COVID-19, Cardiovascular health, Self-care, Theory of planned behavior

## Abstract

**Background:**

The COVID-19 virus has had wide-ranging effects on all healthcare systems and a direct impact on all areas of human life in all countries around the world. Therefore, it is necessary to take preventive actions to reduce the prevalence and severity of the complications associated with this disease. The purpose of this study was to explain the dimensions of adopting general self-care behaviors (mask-wearing, social distancing, hand hygiene, and home quarantine) for preventing COVID-19 based on the theory of planned behavior (TPB) in cardiovascular patients.

**Methods:**

This was a descriptive-analytical study conducted with the participation of 420 patients referring to health and treatment centers of Ahvaz, southwest of Iran, in 2022. Sampling was done using a non-random (convenience) method. The data collection tool was a questionnaire containing items addressing demographic characteristics, questions related to the TPB, and questions dealing with the adoption of everyday self-care behaviors against contracting COVID-19. Data were analyzed using descriptive and inferential statistical methods (prevalence, mean, standard deviation, Pearson’s correlation coefficient, and linear regression) in SPSS version 25.

**Results:**

The results of this study showed that the rate of adoption of self-care behaviors against COVID-19 among cardiovascular patients was moderate. The results also showed that among the constructs of the TPB, Perceived behavioral control, Subjective norms, and Perceived behavioral intention were the most important predictors of adopting self-care behaviors among cardiovascular patients with a change variance of 46%.

**Conclusions:**

The results of the present study have implications for health and treatment policy makers as well as planners of educational and behavioral interventions aimed at promoting the adoption of self-care behaviors against COVID-19. In this respect, managing and institutionalizing desirable behaviors among cardiovascular patients could be beneficial from economic, social, and health-related aspects.

## Introduction

Epidemics are among the biophysical hazards that have historically threatened humanity [1]. One of the most important and dangerous epidemics in human history was identified in Wuhan, China in December 2019, which quickly spread all over the world. The cause of this disease was a virus from the coronavirus family, namely SARS-Cov-2, officially named COVID-19 by the World Health Organization [2]. The United Nations has described COVID-19 as a huge social, human, and economic crisis whose complications will affect even developed countries, if not stopped [3, 4]. The COVID-19 disease is associated with severe complications including thrombotic complications, cardiac and ischemic dysfunction, acute coronary syndrome, acute kidney failure, gastrointestinal symptoms, damage to liver cells, diabetic ketoacidosis, neurological diseases, eye symptoms, and skin complications [5].

In addition to being independently fatal, cardiovascular diseases can threaten a person’s life by laying the groundwork for other environmental and genetic pathogenic factors [6]. According to previous studies, the prevalence of cardiovascular diseases in patients with SARS and MERS has been reported to be 10% and 30%, respectively [7, 8]. Initial reports in China showed that cardiovascular disease and its risk factors such as high blood pressure and diabetes were common comorbidities in patients with COVID-19 [9]. The available studies indicate that in the Eastern Mediterranean and the Middle Eastern countries including Iran, cardiovascular diseases are considered a major health problem whose dimensions are increasing rapidly [10]. In Iran, cardiovascular diseases are the first cause of death, and their case fatality rate has been estimated to have an upward trend in the country [11]. According to a report by the World Health Organization, 41.3% of all deaths in Iran in 2000 were caused by cardiovascular diseases, and this rate is expected to reach 44.8% by 2030 [10, 12].

Given the pervasive and uncontrollable spread of COVID-19 on the one hand and the need to adopt preventive approaches on the other, the most basic and necessary step in designing cognitive-behavioral interventions aimed at controlling and reducing the spread of COVID-19 would be evaluating and identifying the variables that determine preventive behaviors against this disease [13]. Some researchers have used health behavior theories to recognize and explain these determinants, such as the health belief model (HBM) [14, 15], Protection Motivation Theory (PMT [16], and theory of planned behavior (TPB) [17, 18]. The use of behavior change models/ theories to determine the effective factors in persuading people to accept preventive behaviors is considered an economical and reasonable approach [19]. The theory of planned behavior (TPB) is one of the most famous psychological models developed by Ajzen and Fischbein to study the psychological factors affecting health behaviors [19]. According to TPB, it is assumed that the main determinant of behavior is intention. Intention includes the process of thinking and deciding about the possibility of performing a behavior, which is influenced by the three constructs of attitudes toward behavior, subjective norms, and perceived behavioral control. The type of behavior and social, cultural, and psychological characteristics of the audience determine the impact of these constructs [19]. The efficiency and effectiveness of the TPB in describing and predicting a wide range of health behaviors have been confirmed. This includes, inter alia, high-risk sexual behaviors, especially chronic disease prevention behaviors, and SARS prevention behaviors [20]. Therefore, the TPB has the potential capacity to explain the factors affecting health behaviors and the development of cognitive-behavioral interventions [21].

Most theory-based studies in the field of predicting the prevention behaviors of COVID-19 have targeted the general public and professionals. According to researchers’ studies, no study has been conducted among cardiovascular patients. Therefore, the present study was conducted to determine the predictors of adopting preventive behaviors against COVID-19 based on the constructs of the TPB in a population of cardiovascular patients.

## Methods

### Study design

This was a descriptive-analytical cross-sectional study conducted in 2022. It aimed to investigate the factors predicting self-care behavior during the COVID-19 epidemic based on the constructs of the TPB among cardiovascular patients referring to treatment and health centers of Ahvaz, southwest of Iran.

### Study setting and sites

Ahvaz is a metropolis in the center of Khuzestan province, southwest of Iran. This study was conducted in the Gamma Scan Clinic of Hilal and the Nuclear Medicine Center of Golestan Hospital in Ahvaz.

### Study sampling

The number of participants in the study was 420. The sample size was estimated to be 384 people based on Cochran’s formula, considering a proportion of 50%, a confidence level of 95%, and a relative error of 0.05. $x = \frac{{{z^2}pq}}{{{d^2}}}$

Z = 1.96; *p* = q = 0.5; d = 0.05

Due to the epidemic, the patients had concerns about going to clinics; thus, a 10% attrition rate was assumed, and the final sample size was increased to 420 people.

To perform the sampling, the researchers visited the Helal Gamma Scan Clinic and the Nuclear Medicine Center of Golestan Hospital in Ahvaz. These are the two main centers where the majority of patients from different cities of Khuzestan province visit in order to receive specialized and sub-specialized treatments. Sampling was done using a non-random (convenience sample) due to difficult access to patients meeting the inclusion criteria of our study and the limited number of these patients.

The convenience sampling method is susceptible to bias since the researcher selects the sample based on convenience rather than equal probability. Therefore, convenience samples never result in a statistically balanced selection of the population, which leads to sampling bias. To reduce this bias, we tried to distribute the research questionnaire on different days and at different times to select participants with characteristics similar to those of the target population.

### Inclusion and exclusion criteria

In order to be eligible to participate in the study, the participants had to: be 18–65 years of age, have cardiovascular disease, give informed consent to participate in the research, not have COVID-19 (based on self-report), and have the ability to communicate in Farsi. The exclusion criteria were: unwillingness to participate in the study.

### Measurement instruments

The data collection tool of this study was a researcher-made questionnaire containing four sections. The first section addressed the patient’s demographic profile. The second section was devoted to awareness questions (*n* = 12). For example, one item read: “People may be infected with COVID-19 but have no symptoms” which was scored either 0 or 1 depending on the wrong or correct answer given, respectively. The third section included questions dealing with various constructs of the TPB as follows: Attitude towards risk (including 5 questions with a range of 5–25), attitudes towards prevention (including 6 questions with a score range of 6–30), subjective norms (including 7 questions with a score range of 7–35), perceived behavioral control (including 6 questions with a score range of 6–30), behavioral intention (including 6 questions with a score range of 6–30). All constructs of TPB were scored based on a 5-point Likert scale (completely agree: 5, agree: 4, have no opinion: 3, disagree: 2, and completely disagree: 1). The fourth section included 6 questions related to self-care behaviors (with a score range of 6–30) such as wearing masks, social distancing, hand hygiene, and home quarantine which were scored based on a 5-point Likert scale (never: 0, rarely: 1, sometimes: 2, often: 3, and always: 4). It should be noted that the final score of every participant in all constructs was estimated out of 100, and the scores were conventionally divided into three categories: weak (0–50), moderate (51–75) and good (76–100).

### Validity and reliability of the questionnaire

The initial questions of the questionnaire were developed based on relevant articles and the safety instructions against the COVID-19 disease. Face and content validity were used to evaluate the validity of the questionnaire. To qualitatively assess the face validity of the questionnaire, its initial format was sent to 10 people who were experts in different fields (including five experts in health education and promotion, two in statistics and epidemiology, two in infectious diseases, and two in research, design, and development of the questionnaire). All of them were sufficiently familiar with COVID-19. The questions were examined in terms of level of difficulty, degree of inadequacy, ambiguity of phrases, or existence of insufficiency in the meanings.

Also, the Item Impact Method was used to check the face validity quantitatively. Therefore, 20 patients were asked to indicate the importance of each questionnaire item in a 5-point Likert scale from 1 (not at all important) to 5 (completely important). After calculating the item impact scores, only questions with a score higher than 1.5 remained, and the questionnaire was modified accordingly.

In the qualitative content validity review, eight experts were asked to provide their corrective opinions on each item after a detailed study of the questionnaire items (following the grammar, using appropriate words, placing the items in their proper place, and appropriate scoring). To determine content validity quantitatively, the Content Validity Ratio (CVR) and Content Validity Index (CVI) were used. According to the findings obtained from the opinions of eight experts; CVR and CVI of the whole questionnaire were obtained as 0.87 and 0.91, respectively.

The reliability of the questionnaire was measured by running the Cronbach’s alpha test on 30 patients who were similar to the study population in terms of demographic characteristics. The Cronbach’s alpha value of the whole questionnaire was 0.72, and therefore the reliability of the tool was confirmed.

### Data analysis

The data was imported into SPSS version 22 and analyzed using descriptive statistics, such as frequency, mean, and standard deviation. The normality of data distribution was confirmed by the Kolmogorov–Smirnov test, and inferential statistics, such as Pearson’s correlation coefficient test was used because regression analysis allows an understanding of the strength of relationships between variables. Statistical measurements such as R-squared/adjusted R-squared, and regression analysis show how much of the total variability in the data is explained by the model, and regression analysis indicates what predictors in a model are statistically significant or not. Therefore, in linear regression (stepwise) analysis, Self-care behaviors against contracting COVID-19 were considered the dependent variable, while Awareness, Attitudes, Subjective norms and, Intention were considered independent variables. In all statistical analyses, the significance level was set at 0.05.

## Results

This study included 420 patients with an average age of 54.27 ± 12.49, of whom, 162 (38.6%) were male and 258 (61.4%) were female. An overview of the demographic characteristics of the participants is given in Table 1.

**Table 1:** Demographic characteristics of cardiovascular patients (*N* = 420)

Variable		N/ Mean	(%)/SD
Age		54.27	12.49
Gender	Man	162	38,57%
	Female	258	61.43%
Educational attainment	Illiterate	126	30%
	Elementary	98	23%
	Middle school	50	11.9%
	High school diploma	70	17%
	University degree	76	18.1%
Recruitment status	Housewife	266	63.3%
	Employee	57	13.6%
	Self-employed	26	6.2%
	Retired	44	10.47%
	Unemployed	27	6.42%
Receiving training related to COVID-19	Yes	357	85%
	No	63	15%

The participants’ mean score of various dimensions of the constructs of TPB in relation to self-care against COVID-19 was at a moderate level. The most challenging constructs were Awareness (63.36 ± 12.63), followed by Subjective norms (66.66 ± 15.09), Perceived behavioral control (73.67 ± 17.72), and Behavioral intention (75.43 ± 10.36). The mean score of self-care behaviors in relation to COVID-19 was moderate (68.17 ± 13.02) (Table 2).

**Table 2:** Mean scores of the constructs of TPB and self-care behaviors against COVID-19

Statistical index desired variable	Mean	Standard deviation	Maximum	Minimum
Awareness	63.36	12.63	100	8.33
Attitudes	83.21	13.44	100	20
Subjective norms	66.66	15.09	85.71	28.57
Perceived behavioral control	73.67	17.72	100	20
Intention	75.43	10.36	94.29	20
Self-care behaviors against COVID-19	68.17	13.02	96.67	40

Pearson’s correlation coefficient showed a positive and significant relationship of the constructs of the TPB and the gender of the participants with the rate of adoption of preventive self-care behaviors against COVID-19. Other correlation coefficients of the constructs of the TPB and individual factors in connection with adopting self-care behaviors against COVID-19 are shown in Table 3.

**Table 3:** Pearson’s correlation coefficient between demographic factors and the constructs of TPB in relation to self-care behaviors against COVID-19

		Age	Gender	Awareness	Attitudes	Subjective norms	Perceived behavioral control	Intention	Self-care behaviors
Age	r								
	*p*								
Gender	r	0.047	1						
	*p*	0.412							
Awareness	r	0.050	0.093	1					
	*p*	0.391	0.112						
Attitudes	r	0.058	0.166^**^	0.210^**^	1				
	*p*	0.317	0.004	0.000					
Subjective norms	r	0.034	0.148^**^	0.187^**^	0.706^**^	1			
	*p*	0.553	0.010	0.001	0.000				
Perceived behavioral control	r	0.060	0.075	0.046	0.240^**^	0.405^**^	1		
	*p*	0.297	0.192	0.432	0.000	0.000			
Intention	r	0.083	0.091	-0.069	0.217^**^	0.375^**^	0.500^**^	1	
	*p*	0.148	0.111	0.241	0.004	0.000	0.000		
Self-care behaviors	r	0.030	0.116^*^	0.042^**^	0.189^**^	0.314^**^	0.459^**^	0.647^**^	1
	*p*	0.630	0.043	0.000	0.001	0.000	0.000	0.000	

**. Correlation is significant at the 0.01 level (2-tailed)

*. Correlation is significant at the 0.05 level (2-tailed)

Regression analysis and the Stepwise method were used to determine the predictive power of the behavioral intention to adopt self-care behaviors against COVID-19. In this regression analysis, the constructs of TPB and some demographic factors were taken into account, and based on its results, Perceived behavioral control and Subjective norms were identified as the final predictors of changes in the rate of adopting self-care behavioral intention and prevention from COVID-19. These variables could explain about 28% (R2 = 0.278) of the changes in the behavioral intention to adopt self-care behaviors in connection with the prevention of COVID-19. (Table 4).

**Table 4:** Multivariate regression analysis for predicting patients’ self-care behavioral intention

Criterion variable	Predicting variables	Correlation(R)	Coefficient of explanation(R^2^)	Adjusted coefficient of explanation Adjusted(R^2^)
Constructs of the TPB	Perceived behavioral control	0.497	0.247	0.244
	Perceived behavioral control and Subjective norms	0.527	0.278	0.272

Regression analysis and stepwise method were also used to determine the predictive power of adopting self-care behaviors against COVID-19. In this structural regression analysis, the TPB and some demographic factors were taken into account, and based on the results, perceived behavioral intention and perceived behavioral control were identified as the final predictors of changes in the rate of adopting self-care behaviors and preventing COVID-19 infection. These variables could explain about 46% (R2 = 0.464) of the changes in adopting self-care behaviors in connection with the prevention of COVID-19. (Table 5).

**Table 5:** Multivariate regression analysis for predicting patients’ self-care behavior

Criterion variable	Predicting variables	Correlation(R)	Coefficient of explanation(R^2^)	Adjusted coefficient of explanation Adjusted(R^2^)
Constructs of the TPB	Intention	0.669	0.447	0.445
	Intention and Perceived behavioral control	0.681	0.464	0.460

## Discussion

This study aimed to investigate the factors predicting the adoption of self-care behaviors against COVID-19 among cardiovascular patients based on the TPB. The results of the study generally showed that TPB constructs have a significant effect in predicting preventive behaviors from COVID-19 in cardiovascular patients. In addition, perceived behavioral control and subjective norms play a role in predicting the intention to adopt self-care behaviors, as well as behavioral intention and perceived behavioral control in predicting preventive behaviors from COVID-19. In line with the results of this study, other studies have also confirmed the effectiveness of this theory [22, 23] especially the two constructs of subjective norms and perceived behavioral control in explaining the preventive behaviors of COVID-19 [24].

The outcome of this research includes two statistical models for predicting the intention to adopt self-care behaviors and directly predicting the adoption of self-care behaviors. The first statistical model consists of two variables, namely perceived behavioral control and subjective norms, which can explain 28% of the intention to adopt self-care behaviors related to COVID-19. The second statistical model includes two variables, namely perceived behavioral intention and perceived behavioral control, which can explain nearly 46% of adoption of self-care behaviors related to COVID-19. In previous research, the effectiveness of the theory of planned behavior in explaining human behavior has been widely confirmed. For instance, in Yahaghi et al.‘s research, theory constructs predicted 67% of behavioral intention [25], and Sadri et al.‘s study predicted 34% of changes in vaccination behavior and 37% of changes in vaccination intention [26], these differences can be caused by differences in influential demographic variables (such as age, gender, and having a disease), methodological approaches, and periods of data collection on adopting behavior intention.

One of the important findings of the study was that the construct of perceived behavioral control had a significant positive effect on behavioral intention and preventive behaviors from COVID-19. So that the behavior of patients to follow preventive behaviors related to COVID-19 is influenced by their perceived behavioral control, which was in line with the findings of previous research [23, 27]. A meta-analysis of 19 studies from 11 countries showed that perceived behavioral control has a strong effect on the intention and behaviors leading to the prevention or reduction of COVID-19 infection [23]. Given that perceived behavioral control is strongly influenced by perceived barriers; therefore, identifying various personal, social, and environmental barriers to adopting preventive behaviors through strategies such as brainstorming and helping to find suitable solutions can lead to an increase in preventive performance. Also, since self-efficacy is an important component of perceived behavioral control, using self-efficacy promotion strategies is effective in promoting preventive behaviors.

The next important finding in our study was the positive and significant relationship between the construct of subjective norms and behavioral intention. Also, this construct was proposed as an important determinant in predicting behavioral intention in model number 1. This finding was consistent with previous studies [23, 28, 29]. This is even though, in Alshagrawi’s study, the subjective norm structure was not a significant predictor for vaccination intentions [30]. family and friends; treatment staff, effective role models, officials, and community leaders have a strong role in encouraging and influencing people’s intention to adopt preventive behaviors against COVID-19; therefore, effective interventions to improve preventive behaviors should include the involvement of influential figures (such as religious leaders, athletes, and artists) to encourage patients to follow prevention protocols.

Another important finding of this study was the positive and significant relationship between attitude with behavioral intention and the adoption of self-care behaviors. Also, attitude has a positive and significant relationship with perceived behavioral control and subjective norms. As mentioned in the introduction section, according to the TPB, the adoption of self-care behaviors is influenced by behavioral intention, and the intention is influenced by the three constructs of attitude, subjective norms, and perceived behavioral control [31]. In line with our results, Prasetyo et al. reported that the intention to pursue and perform preventive behaviors, along with the constructs of social norms and perceived behavioral control are predictors of actual preventive behaviors against COVID-19 [29]. Also, Adiyoso and Wilopo’s study showed that understanding the risk of COVID-19 disease affects improving social distancing through the improvement of attitude toward prevention, subjective norms, and perceived behavioral control [24]. In this way, patients who have a positive attitude towards self-care behaviors and preventive ways of disease and also understand the risk of disease seriously, will have more intention to adopt these behaviors. In this study, we evaluated the attitude towards the ways of prevention and understanding the risk of the disease. Although the relationship between attitudes with model constructs was positive and significant, in this study, the attitude construct did not contribute to the prediction of the intention and self-care behaviors against COVID-19. Of course, it should be noted that the adoption of self-care behaviors in cardiovascular patients can be affected by some non-behavioral environmental factors. These include the availability of facilities and resources to prevent the spread of COVID-19, the high cost of protective and disinfectant agents in stores, the strategies adopted, etc. Further studies in this field are expected to inform society and officials to adopt useful policies and implement effective interventions. In this way, it will be possible to increase the adoption of self-care and preventive behaviors against COVID-19. On the other hand, to strengthen the attitude regarding the adoption of preventive behaviors, it is recommended to provide real statistics about the number of people infected with COVID-19, the number of deaths due to COVID-19, correct feedback about the consequences of the disease by the media, especially in groups at risk such as cardiovascular patients.

Based on the results of this research, the mean score of adopting self-care behaviors of cardiovascular patients against COVID-19 was 68, which does not seem to be a desirable level considering the high prevalence of COVID-19 around the world and in Iran. Although a similar study has not been conducted to compare self-care behaviors against COVID-19 in cardiovascular patients, one of the most important factors that highly affects the spread of COVID-19 is people’s decision to take preventive measures such as social distancing, home quarantine, and compliance with hygienic measures in their daily activities [32]. Also, human psychological and behavioral factors play a pivotal role in the management of global epidemics such as COVID-19. Therefore, it is important to assess the behavioral and psychological responses to this situation and determine the degree of association of perceived risk with the adoption of protective behaviors to facilitate disease minimization strategies [33]. It is also necessary to examine the psychological factors affecting the occurrence of preventive health behaviors. More particularly, to control the spread of COVID-19, it is necessary to acquire sufficient information about health beliefs and behaviors related to the risk of COVID-19 [34].

To enhance the effectiveness of interventions aimed at promote the adoption of self-care behaviors against COVID-19 in cardiovascular patients, it is necessary to identify the factors that determine compliance with preventive and self-care behaviors among these patients. In the present study, knowledge, attitude, subjective norms, perceived behavioral control, and behavioral intention were positively correlated with the adoption of preventive self-care behaviors. This finding is consistent with the results of previous studies [13, 35] and can be justified by the widespread epidemic of COVID-19 all around the world. It should be noted that in this study, demographic factors of age, educational attainment, and occupation did not correlate with the adoption of preventive self-care behaviors.

According to our results, perceived behavioral control was the strongest and most effective variable as far as the intention to adopt self-care behaviors for COVID-19 was concerned. It can alone predict the intentions to adopt self-care behaviors against COVID-19 in cardiovascular patients with a change of variance of about 24%. Perceived behavioral control is a concept that is related to a person’s understanding of their resources regarding the skills, time, and money needed to do a specific task. In other words, it refers to a person’s belief that they can control the situation and manage the resources necessary to do a certain task [36]. In general, people are more inclined to perform a behavior that is perceived as more feasible [37].

The subjective norms construct is the second construct of the TPB that could describe the intention to adopt self-care behaviors against contracting COVID-19. Specifically, in Iran, Subjective norms should be considered the main factor affecting behavioral intention. This is because even during the peak of the spread of COVID-19, there were no strict restrictions and quarantine in Iran, and health authorities only asked citizens to observe social distancing and health protocols. However, it should be noted that people’s behaviors are highly influenced by social pressures when it comes to complying with preventive behaviors such as social distancing, wearing masks, and frequent hand washing to avoid public blame or to please important others in their lives [38].

In this study, the constructs of behavioral intention and perceived behavioral control of the TPB were predictors of adopting self-care behaviors against COVID-19, with a change of variance of about 46%. In the meantime, the construct of behavioral intention is considered the strongest predictor of adopting preventive behaviors against COVID-19. Intention is a mental state that entails an obligation to act in the future, which includes the underlying motivation or obligation to act. Intention includes mental activities such as planning and foresight [39, 40]. The transition from behavioral intention to adopting behavior is strongly influenced by perceived barriers. Therefore, identifying various personal, social, and environmental barriers and promoting the enabling factors for observing preventive behaviors can enhance preventive performance.

Following the construct of behavioral intention, the construct of Perceived behavioral control is the second construct of the TPB that could explain the adoption of self-care behaviors against contracting COVID-19. With regard to the effect of perceived behavioral control, it can be argued that the spread of the COVID-19 virus in society will decrease as more people have the ability to regulate and maintain their behavior according to available resources, opportunities, and conditions governing the atmosphere of COVID-19 in society. This includes, for example, postponing their daily routines and not leaving home. However, transformation of intention into behavior also depends on norms and people’s evaluation of the consequences of their actions [41]. This perceived behavioral control means a person’s perception of their ability to promote and perform healthy behavior. In other words, the level of motivation and ability of individuals to continue health behaviors against the spread of the COVID-19 virus depends on their perceived behavioral control. Also, since self-efficacy is an important component of perceived behavioral control, using self-efficacy promotion strategies such as setting goals, turning general goals into specific and staged goals, observing behavioral patterns, and verbal persuasion are effective in promoting preventive behaviors. Therefore, enhancing this perception of critical conditions and preventing the normalization of conditions is very important.

## Conclusion

According to the results of this study, the theory of planned behavior can predict behavioral intention and the adoption of self-care behavior in cardiovascular patients with a variance of about 27% and 46%, respectively. Perceived behavioral control, Subjective norms, and Behavioral intention were among the strongest predictors of adopting self-care behaviors against COVID-19. These findings have implications for planning, designing interventions, and formulating appropriate strategies for adopting self-care behaviors against COVID-19 in the first place, and controlling and preventing the effects of the spread of the COVID-19 epidemic on health.

### Limitations and strengths

Due to the cross-sectional design of the study, it was difficult to determine the cause-and-effect relationship. Therefore, this type of study should be repeated in larger communities, and clinical and interventional studies should be conducted. Another limitation of the study was that performance measurement, was based on self-report. Of course, by stating the objectives of the study and assuring the patients of the confidentiality of their information, an attempt was made to reduce this limitation. Finally, more extensive studies with more samples from different urban and rural areas and different provinces are suggested to increase the generalizability of the results.

### Application of clinical practice

The following points provide a suitable framework for applying the TPB in clinical practice to better understand the factors affecting the self-care behaviors of cardiovascular patients against COVID-19:

1. By identifying predictors of self-care behavior, clinicians can develop targeted strategies to improve patient outcomes and reduce the risk of complications.
2. Targeted Intervention Strategies: The TPB can be used to identify effective intervention strategies for adopting preventive self-care behaviors against COVID-19 in cardiovascular patients.
3. Patient education and empowerment: With the help of educational programs based on this theory, cardiovascular patients can be empowered to actively participate in their health management (Through attention to attitudes, subjective norms, and perceived behavioral control related to self-care).
4. Education of health care providers: By training health care providers using this theory, they can be equipped with the skills to identify subjective norms, attitudes, and determinants of perceived behavioral control in cardiovascular patients and ultimately improve their self-care behaviors.
5. Tailored Communication Strategies: The insights of this theory can be used to create communication strategies to adopt, maintaining, and promote self-care behaviors in cardiovascular patients against COVID-19.
6. The identification of factors predicting self-care behavior against COVID-19 can guide the allocation of resources and healthcare personnel to better meet the specific needs of cardiovascular patients, ultimately improving overall care delivery.

## Data Availability

The datasets used and/or analyzed during the current study are available from the corresponding author on reasonable request.

## Abbreviations

TPB: Theory of Planned Behavior
SPSS: Statistical Package for Social Sciences
WHO: The World Health Organization
HBM: Health Belief Model
PMT: Protection Motivation Theory
CVR: Content Validity Ratio
CVI: Content Validity Index
